# Which Are the Most Relevant Questions in the Assessment of Outcome after Distal Radial Fractures?

**DOI:** 10.1155/2015/460589

**Published:** 2015-12-30

**Authors:** Donald Buchanan, D. Prothero, J. Field

**Affiliations:** ^1^Department of Trauma & Orthopaedics, Good Hope Hospital, Rectory Road, Sutton Coldfield B75 7RR, UK; ^2^Medical Statistician, Avon IM&T Consortium, NHS Bristol, South Plaza, Marlborough Street, Bristol BS1 3NX, UK; ^3^Department of Trauma & Orthopaedics, Cheltenham General Hospital, Sandford Road, Cheltenham GL53 7AN, UK

## Abstract

A study was designed to determine which wrist scoring system best correlates with patient satisfaction and which individual variables predict a satisfactory outcome. We looked at forty-five females and 5 males with wrist fractures at 12 weeks after injury and compared their level of satisfaction with various respected outcome measures. The mean age was 66 years. Multivariate regression analysis was carried out using a statistical software package. Patient satisfaction correlated best with the MacDermid, Watts, and DASH scores. The variables in these scoring systems that predicted satisfaction were pain and ability to perform household chores or usual occupation, open packets, and cut meat. The four most important questions to ask in the clinic following wrist fractures are about severity of pain and ability to open packets, cut meat, and perform household chores or usual occupation. This may provide simple and more concise means of assessing outcome after distal radial fractures. Level of evidence is level 4.

## 1. Introduction

Outcome assessment is important in evaluating the efficacy of different treatment strategies and improving patient care. Health care providers use outcome assessment to assess the quality and value of care. Some of the more useful scoring systems for the assessment of outcome following fractures of the distal radius are set out below.

- Gartland Jr. and Werley [1] introduced a demerit score which combines the subjective evaluation of pain, limitation of motion, and disability with the objective evaluation of deformity, range of motion, presence of complications, and radiographic changes of arthritis. Scores range from 0–2 (excellent) to more than 21 (poor). This score was widely used despite its lack of validation studies.
- Sarmiento et al. [2] modified the Gartland and Werley score by adding grip strength to the objective evaluation; a grip strength of 60% or less of the opposite side scored 1 point.
- The Mayo Wrist score [3] has two subjective parameters, pain and functional status, each of which is awarded 25 points. Each objective parameter, range of motion, and grip strength is awarded 25 points. An excellent score is 90 to 100 and a poor score is less than 65. Neither the Sarmiento nor the Cooney score has been validated.

The above scores rely on formal clinical assessment as well as radiological parameters [1]. An outpatient visit is required and is thus costly and time consuming. What is measured by traditional functional metrics does not always translate to outcome desired by the patient, healthcare provider, or society. This has emphasised the need to use patient reported outcome metrics in the assessment of upper extremity disease.

- The Patient-Rated Wrist Evaluation [4] provides a brief, reliable, and valid measure of patient-rated pain and disability; the score ranges from 0 (no pain or disability) to 100.
- The Disability of Arm, Shoulder, and Hand (DASH) Score [5] is a self-administered, region specific quality of life instrument which contains thirty questions, twenty-one of which relate to upper extremity functional activities and two of which relate to upper extremity pain. The score ranges from 0 (no disability) to 100 (severe disability). DASH has good validity, reliability, and responsiveness [6, 7]. Patients take 10 to 15 minutes to complete the questionnaire and the administrator requires 10 minutes to compute the score making it a time consuming outcome instrument. Beaton et al. [8] produced the Quick DASH an abbreviated version of the DASH score which has eleven questions. This has been validated by several authors including Gummesson et al. [9] and Matheson et al. [10].
- The hand function score [11] is a subjective hand function scoring system based on twenty-five commonly performed activities of daily living. The range of the total score is from 25 to 100 (the worst score). The authors showed a positive correlation between the hand function score on admission and both severity of injury and length of time off work but the score has not been validated.

In a busy fracture clinic it would be helpful to have a quick way of assessing outcome after distal radial fractures; it would be helpful to define a few questions that could be used to rapidly assess these patients. A study was designed to determine which variables predict patient satisfaction with the outcome after distal radial fractures.

## 2. Material and Methods

Over the period of 1 year, fifty adult patients who had sustained a distal radius fracture were evaluated during follow-up in a district general hospital fracture clinic. Intra-articular, extra-articular, and closed as well as open distal radius fractures were included. Informed consent was obtained before inclusion in the study. Patients below the age of 18 years and those not agreeing to participate were excluded. There were forty-five women and 5 men (Table 1) with an average age of 66 years (range of 18–93 years). Most of the fractures were classified as Frykman I to Frykman III (Table 2). Fractures were fixed either with K wires or a plate through a volar approach. All patients, including those in whom the fracture was fixed, were immobilised in a below elbow plaster of Paris cast for six weeks and reviewed six weeks after removal of the plaster. None of the patients were treated with an external fixator.

All participants completed a comprehensive questionnaire which incorporated all the questions included in the six scoring systems considered in this study. The patients recorded their satisfaction with the outcome of treatment on a 10-centimeter-long visual analogue scale [12] which ranged from not at all satisfied (0) to completely satisfied (10). Clinical examination focused on the parameters defined in the above scoring systems. Grip strength was measured using a Jamar dynamometer [13].

### 2.1. Statistical Methods

The relationship between the patient satisfaction score and the total score from each of the six scoring systems was initially examined by computing the Pearson correlation coefficient. A multiple regression analysis was then undertaken to examine the relationship between the score for each system (including patient satisfaction) and the overall scores for the other 6 systems. The relationship between the overall scores for each system was also examined in relation to the individual components included in each of the six systems. In each case, the regression model was chosen such that the “fit” was not significantly improved through inclusion of additional variables but for which the “fit” was significantly worse if any of the variables included were removed.

The stepwise multiple regression analysis of the overall score for each system on the individual components within that system was used to identify the key variables within each individual system. The proportion of the total variation explained by each component was determined in decreasing order of magnitude. The key variables in each system were defined as those which explained the highest proportion of the total variation and which together accounted for at least 95% of the total variation.

An overall set of key variables was then created based on all of the variables which featured in at least one of the individual scoring systems. Stepwise multiple regression analysis was then undertaken for each scoring system using the overall set of key variables as independent variables in order to identify a small subset of variables which contributed most in explaining the overall variation within the scoring systems.

### 2.2. Sample Size Calculation

The computation of sample size was arrived at on the basis of the correlation between patient satisfaction and the total scores generated by each system.

When the true correlation coefficient is zero, it can be shown that the statistic √*r*(*n* − 2)/(1 − *r* ^2^) has a *t* distribution with *n* − 2 degrees of freedom where *r* is the observed correlation coefficient and *n* is the number of observations within the sample. The correlation is significantly different from zero at the five percent significance level if the above statistic is greater than or equal to *t*_.05_ *n* − 2. It can be shown that for an observed correlation coefficient of 0.35 to be significantly different from zero at a 5% level of significance and with a power of 80% then a sample size of 49 is required.

## 3. Results

**Figure 1:**
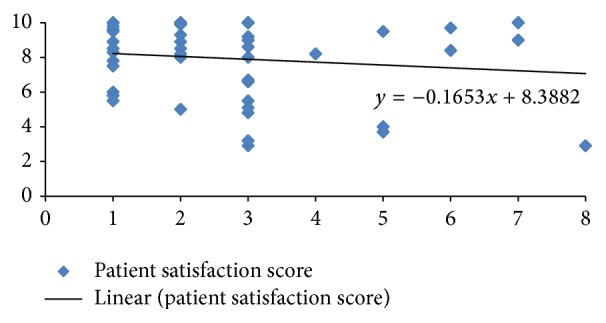
Plot of patient satisfaction score (*y*-axis) versus Frykman class (*x*-axis).

The mean satisfaction score was 7.9. Eleven of the patients were completely satisfied with the outcome of the treatment. There was poor correlation between the patient satisfaction score and the Frykman classification of the fracture pattern (Figure 1). Spearman's rho coefficient for the relationship between patient satisfaction and Frykman score was 0.09 (not significant).

When the correlation (Table 3) between each pair of scoring systems (based on the overall score associated with each of the 6 systems) and the patient satisfaction score was examined there was a highly significant correlation (*p* < 0.01) between the scores for the 6 systems. There was also a highly significant correlation between patient satisfaction and the MacDermid (−0.39) and Watts (−0.36) systems. The correlation between the patient satisfaction and the DASH system (−0.28) was only moderately significant (*p* < 0.05). No significant correlation (*p* > 0.05) was shown between patients' satisfaction and the Gartland and Werley and Sarmiento and Cooney systems.

The summary of the results of undertaking a multiple regression analysis of the overall score for each individual system on the overall scores for all other systems (Table 4) explains the extent to which the scoring systems (and patient satisfaction) contribute to the other scoring systems and may indicate the degree of overlap. In each case the table highlights the “best” combination of explanatory variables; that is, the inclusion of any additional variables will not significantly improve the fit of the model.

The MacDermid and DASH scoring systems are the only systems that feature as explanatory variables for the patient satisfaction score, although together they account for only twenty percent of the total variation. Patient satisfaction featured as an explanatory variable for only 2 of the scoring systems, that is, the MacDermid and DASH scores. The MacDermid, Gartland, and Werley and patient satisfaction all made significant independent contributions in explaining the DASH score, whereas the questions in the Sarmiento, Cooney, and Watts systems contributed nothing further.

From the analysis based on the component variables highlighted (Table 5), four variables, namely, ability to perform usual occupation (including housework), ability to open packets, ability to cut meat, and severity of pain, account for 94% of the variation associated with the MacDermid score, 91% of the Watts score, and 93% of the DASH score. However, these 4 variables account for only 56% of the Gartland and Werley score, 58% of the Sarmiento score, and 26% of the Cooney score. These four variables are the most relevant variables in explaining the overall variation within the six scoring systems.

Furthermore, they explain a high percentage of the variation within the MacDermid, Watts, and DASH systems, that is, the systems which were most highly correlated with patient satisfaction. The two most important variables were pain and ability to cut meat. Work and house work make a similar contribution and may be used interchangeably.

The percentage of the variation in the score (Table 5) explained by each individual variable is substantially lower for the Gartland and Werley and Sarmiento and Cooney systems than for the DASH, Watts, and MacDermid systems. This is not surprising as, with the exception of pain, the variables identified are of a patient assessed functional nature and do not feature within the Gartland and Werley and Sarmiento and Cooney scoring systems.

## 4. Discussion

Patient satisfaction levels were high at three months following the distal radial fracture. A mean value of 80% satisfaction would agree with Abraham Colles original treatise. Data analysis however did not show any significant correlation between patient satisfaction and the Gartland and Werley and Sarmiento and Cooney scoring systems. Since one of the most commonly used evaluation systems is the Gartland and Werley system this may be of some concern; however, these systems for the most part provide an objective evaluation of outcome based on assessments by health personnel. In addition, because Gartland Jr. and Werley [1] and Sarmiento et al. [2] use demerit scoring systems it is unusual to find a poor result even at ten years [14].

The lack of correlation may be because a satisfactory radiological appearance or clinical examination predicts neither good functional outcome nor patient satisfaction [15]. Both Gartland Jr. and Werley [1] and Sarmiento et al. [2] include radiological parameters as part of their scoring systems. More recently Souer et al. [16] showed that following treatment of distal radial fractures there was no significant correlation between pain and radiological parameters such as volar angulation, ulnar variance, articular congruity, and osteoarthritis.

Three of the scores do correlate with patient satisfaction, the MacDermid, Watts, and DASH scores. Of note is the fact that these scoring systems provide a subjective evaluation of function. They provide a better measure of patient satisfaction with the outcome of treatment. The findings in the current study are consistent with those of Souer et al. [16] who showed only moderate correlation between the Gartland and Werley and Cooney and DASH scores. Pain was the main predictor of outcome in these three scores. Souer et al. [16] support the views of MacDermid et al. [17] who argue that comprehensive evaluation of outcome after distal radial fractures requires assessment of objective physical impairment as well as subjective self-rated disability.

There is obviously significant overlap in the questions involved in the 6 assessment methods. The MacDermid score correlates with patient satisfaction as does the DASH score, despite the latter having no question within it concerning satisfaction. The 99.5% of contribution made to the Gartland and Werley by the Sarmiento score is not surprising as the latter was developed from the former and this demonstrates the effectiveness of the method of stepwise regression analysis. The fact that Gartland and Werley and MacDermid scores in conjunction with patient satisfaction accrue 91.3% of the DASH and the Sarmiento, Cooney, and Watts scores contribute nothing further shows the significant level of redundancy in the questions; that is, they are asking similar things.

The current study involved a comparison of six wrist scoring systems only two of which, MacDermid and DASH, have been validated. It shows that four variables, namely, ability to perform usual occupation (including housework), ability to open packets, ability to cut meat, and severity of pain, account for the greatest variation in the six scoring systems. In addition they were the most important in the three questionnaires that correlate most with patient satisfaction (MacDermid, Watts, and DASH). In the clinical setting four questions should be asked in assessing outcome after distal radial fractures. They are as follows. Do you have wrist pain? Can you do your work (including housework)? Can you cut meat? Can you open packets? In ranking importance of the questions, the ability to work and the ability to cut meat are the most important factors. We are currently working on the Distal Radius Assessment Criteria (DRAC) using these 4 questions that could be applied in the clinic situation. The object is to validate this using a larger cohort of patients.

This study is a preliminary study that does have its limitations. It used a small cohort of patients in a district general hospital rather than in level 1 trauma centre. As such most of the injuries were low velocity injuries. Nonetheless, this pilot study indicates that there is the potential to develop a scoring system that is simple, short, and easy to use and may be further investigated.

The Patient Evaluation Measure [18], which is used increasingly in the UK, is a self-administered instrument for outcome assessment of hand and wrist patients; it was validated in a study of scaphoid fracture patients [19] and patients who had carpal tunnel decompression [20]. The PEM was not used in this study as it assesses not only the injury or disease process but also the total care experience.

## Figures and Tables

**Table 1:** Demographics of study group (*n* = 50).

		Number of patients
Sex	Males	5
	Females	45

Handedness	Right	49
	Left	1

Side affected	Right	16
	Left	34

**Table 2:** Fracture pattern on initial postinjury X-rays.

Frykman classification	Number of patients
I	13
II	10
III	17
IV	1
V	3
VI	2
VII	3
VIII	1

**Table 3:** Correlation matrix using Pearson correlation of patient satisfaction with six scoring systems (based on 50 observations).

Scoring system	Patient satisfaction	MacDermid	DASH	Gartland and Werley	Sarmiento	Cooney	Watts
Patient satisfaction	1	−.394 (0.005^*∗∗*^)	−.284 (0.046^*∗*^)	−0.249 (0.081)	−0.26 (0.068)	0.215 (0.133)	−.364 (0.009^*∗∗*^)
MacDermid	−.394 (0.005^*∗∗*^)	1	.949 (0^*∗∗*^)	.702 (0^*∗∗*^)	.710 (0^*∗∗*^)	−.368 (0.009^*∗∗*^)	.935 (0^*∗∗*^)
DASH	−.284 (0.046^*∗*^)	.949 (0^*∗∗*^)	1	.737 (0^*∗∗*^)	.738 (0^*∗∗*^)	−.397 (0.004^*∗∗*^)	.903 (0^*∗∗*^)
Gartland and Werley	−0.249 (0.081)	.702 (0^*∗∗*^)	.737 (0^*∗∗*^)	1	.997 (0^*∗∗*^)	−.565 (0^*∗∗*^)	.787 (0^*∗∗*^)
Sarmiento	−0.26 (0.068)	.710 (0^*∗∗*^)	.738 (0^*∗∗*^)	.997 (0^*∗∗*^)	1	−.579 (0^*∗∗*^)	.798 (0^*∗∗*^)
Cooney	0.215 (0.133)	−.368 (0.009^*∗∗*^)	−.397 (0.004^*∗∗*^)	−.565 (0^*∗∗*^)	−.579 (0^*∗∗*^)	1	−.455 (0.001^*∗∗*^)
Watts	−.364 (0.009^*∗∗*^)	.935 (0^*∗∗*^)	.903 (0^*∗∗*^)	.787 (0^*∗∗*^)	.798 (0^*∗∗*^)	−.455 (0.001^*∗∗*^)	1

Significance levels are in parentheses. The asterisks indicate statistically significant results.

**Table 4:** Summary of stepwise regression analysis of each scoring system on all other scoring systems.

Summary of stepwise regression analysis of each scoring system on all other scoring systems					
Dependent variable	Combination of independent variables explaining a statistically significant (*p* < 0.05) amount of the variation within the dependent variable				Percentage of variation explained
Patient satisfaction	MacDermid	DASH			20.4%
MacDermid	DASH	Watts	Patient satisfaction	Gartland and Werley	94.1%
DASH	MacDermid	Gartland and Werley	Patient satisfaction		91.3%
Gartland and Werley	Sarmiento				99.5%
Sarmiento	Gartland and Werley	Watts			99.5%
Cooney	Sarmiento				32.2%
Watts	MacDermid	Sarmiento			90.7%

**Table 5:** Percentage of variation within each scoring system explained by subsets of variables (*n* = number of variables).

Scoring system								
Variables	*n*	Gartland and Werley	Sarmiento	Cooney	DASH	Watts	MacDermid	Patient satisfaction
Opening packets, cutting meat, work (including housework), worst pain	4	56%	58%	26%	93%	91%	94%	23%
Opening packets, cutting meat, housework, worst pain	4	56%	58%	20%	90%	90%	85%	22%
Opening packets, cutting meat, worst pain, work	4	55%	57%	24%	83%	89%	93%	23%
Work	1	30%	31%	4%	77%	64%	84%	5%
Opening packets	1	47%	49%	24%	57%	76%	58%	1%
Cutting meat	1	45%	47%	17%	60%	76%	70%	7%
Housework	1	30%	29%	12%	74%	50%	56%	2%
Worst pain	1	21%	22%	0%	43%	45%	59%	19%
